# Cases of ergotism in livestock and associated ergot alkaloid concentrations in feed

**DOI:** 10.3389/fchem.2015.00008

**Published:** 2015-02-18

**Authors:** A. Morrie Craig, James L. Klotz, Jennifer M. Duringer

**Affiliations:** ^1^Department of Biomedical Sciences, College of Veterinary Medicine, Oregon State UniversityCorvallis, OR, USA; ^2^Forage-Animal Production Research Unit, United States Department of Agriculture-Agricultural Research ServiceLexington, KY, USA; ^3^Department of Environmental and Molecular Toxicology, Oregon State UniversityCorvallis, OR, USA

**Keywords:** ergot, ergotism, ergovaline, ergotamine, ergocornine, clinical disease, saphenous vein, cattle

## Abstract

Ergot-induced disease in humans was known long before Biblical times and has been the root cause for countless human epidemics spanning from the early fourteenth century to the late sixteenth century. In contrast, many of these same ergot alkaloids have been utilized for their medicinal properties to mitigate migraine headaches and have had indications as anti-carcinogens. Although ergot alkaloids have been used for centuries by humans, basic pharmacokinetic data has not been documented for clinical disease in livestock. Consequently, a threshold dose and accurate dose-response data have yet to be established. Throughout the past several years, new detection techniques have emerged to detect these alkaloids at the parts per billion (ppb) level which has allowed for new efforts to be made with respect to determining threshold levels and making accurate clinical diagnoses in affected animals. This perspectives article provides a critical initial step for establishing a uniform interpretation of ergot toxicosis from limited existing data.

## Introduction

**Figure 1:**
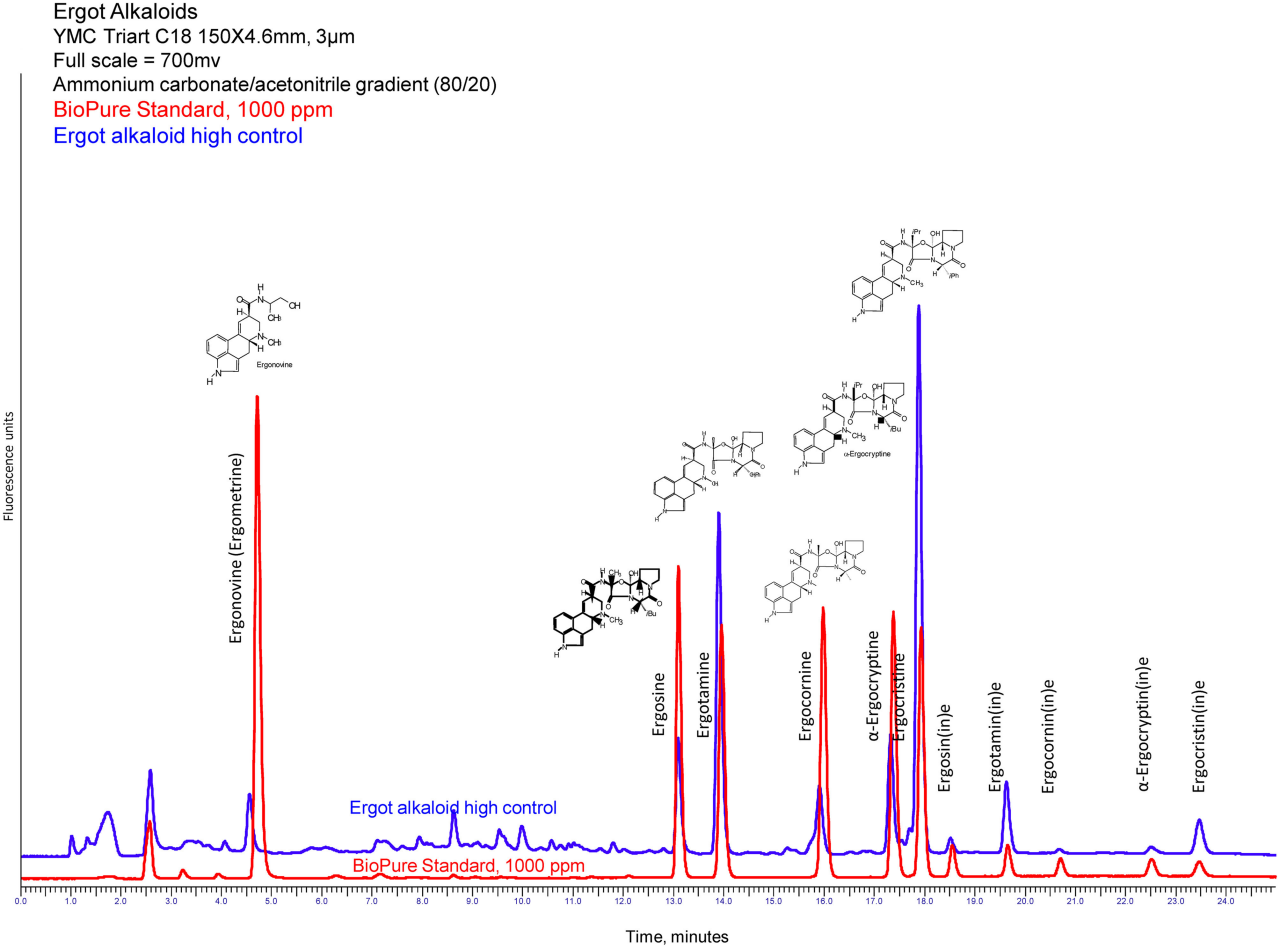
**HPLC fluorescence chromatogram of ergot alkaloids, both pure standards and extracted feed sample containing ergot bodies (Craig et al., 2014)**.

Ergot alkaloids are made as secondary metabolites of fungi. Their production occurs in the sclerotia of several species of the genus *Claviceps*, the most common being *Claviceps purpurea*. These compounds belong to the family of indole alkaloids, one of the ten classes of alkaloids that pharmacologists have defined from plant secondary metabolites. Lysergic acid, a tetracycline ergoline ring system, is a common structure to all of the ergot alkaloids (Krska et al., 2008). The chemical structure of ergotamine, the most commonly found ergot alkaloid and the one responsible for the clinical disease known as ergot, has been documented in a classic text (Matossian, 1989; Bennett and Bentley, 1999; Council for Agricultural Science and Technology, 2003). Other major alkaloids detected in ergot sclerotia include ergocornine, ergocristine, ergosine, and ergonovine, and α-ergocryptine (Krska et al., 2008; Wallwey and Li, 2011; Takach and Young, 2014; also see Figure 1, the chromatogram).

Historically, ergot alkaloids have had a large impact on societies. They are likely the cause for the last of the 10 plagues of Egypt; it is believed that many of the oldest sons succumbed following the opening of grain and storage facilities whose contents were contaminated by the *Claviceps* fungi (Marr and Malloy, 1996). Accounts from the Middle Ages describe “St. Anthony's fire,” a symptom of intense inflammation attributed to human consumptions of food prepared with ergot-contaminated rye. Furthermore, Marie Antoinette's comment, “Let them eat cake, not bread,” most likely surfaced because of ergot contaminated rye bread which was a common food among peasants.

Currently, modern grain cleaning techniques have eliminated ergotism as a human disease, but it remains a significant and devastating disease in the veterinary field primarily affecting sheep, cattle, pigs, and chickens. Clinical signs of ergotism in animals include gangrenous extremities, abortion, convulsions, agalactia, and ataxia (Lorenz and Hoseney, 1979; Richard, 2007; Duringer et al., 2012). Agalactia and the loss of ears, tails, and hooves are common sequelae seen in animals due to vasoconstriction: ergot alkaloids affect the supply of blood to the extremities of the body in addition to acting directly on the central nervous system via the pituitary where they activate the D2 dopamine receptors (Oliver, 2005; Richard, 2012). The tetracyclic ergoline ring of ergot alkaloids is similar to the structure of biogenic amines (Eckert et al., 1978), allowing them to act on the dopaminergic (Larson et al., 1999), serotonergic (Dyer, 1993; Schoning et al., 2001), and α-adrenergic receptors (Oliver et al., 1998; Schoning et al., 2001). Most recently, plant agronomists have shown different varieties within a plant species can portray different “chemotypes” or constitutions different alkaloids (Wallwey and Li, 2011; Panaccione et al., 2012, 2014; Takach and Young, 2014). Sclerotia show significant differences in their total alkaloid content, varying between 0.01 and 0.05% (Krska and Crews, 2008). Patterns that occur in alkaloid production are attributed to an individual strain in a geographic region (Krska and Crews, 2008).

In the United States, there is no regulatory limit defined for ergot alkaloids in grain. In Europe, while there are discussions on establishing legal limits, the European Food Safety Agency (EFSA) at this time has not recommended a limit other than 0.03–3.6 and 0.6 μg/kg b.w. per day for children and adults, respectively (European Food Safety Authority, 2012).

Two types of ergotism have been described: gangrenous (vasoconstrictive) and convulsive (neurological and abortogenic) (da Rocha et al., 2014). Though these two designations exist, the etiology of both is rooted in the vasoconstrictive ability of ergot alkaloids. Associating each alkaloid with a vasoconstrictive measurement (bovine lateral saphenous vein contractile response) has been a key factor in an accurate evaluation of clinical disease and will be discussed in Table 1.

**Table 1:** **Concentration at onset of contractile response, half maximal effective concentration or potency (EC50), and the maximal response or efficacy (EMAX) of ergot alkaloids in bovine lateral saphenous veins^a^**.

**Alkaloid**	**Onset (M)^b^**	**EC_50_ (M)**	**E_MAX_ (%)**	**Ergotamine equivalence level**
Ergovaline	1 × 10^−8^	4.0 × 10^−6^ ± 1.5 × 10^−6^	104.1 ± 6.0	1
Ergotamine	1 × 10^−8^	4.0 × 10^−6^ ± 1.5 × 10^−6^	104.1 ± 6.0	1
Ergonovine	1 × 10^−7^	3.4 × 10^−6^ ± 8.8 × 10^−7^	68.5 ± 4.1	0.1
Ergocristine	1 × 10^−7^	5.6 × 10^−6^ ± 1.3 × 10^−6^	45.5 ± 4.5	0.1
Ergocornine	1 × 10^−7^	4.0 × 10^−5^ ± 2.3 × 10^−5^	57.2 ± 9.9	0.1
α-Ergocryptine	1 × 10^−6^	5.4 × 10^−6^ ± 1.2 × 10^−6^	42.9 ± 4.1	0.01
Lysergic acid	1 × 10^−5^	5.5 × 10^−5^ ± 2.3 × 10^−5^	22.6 ± 4.1	0.001

a *Taken from Klotz et al. (2006, 2007, 2010)*.

b *Expressed as a percent of norepinephrine maximum, which was a 1 × 10^−4^ M norepinephrine reference addition. Onset is normalized to the closest order of magnitude*.

The Endophyte Service Laboratory (ESL) (Craig et al., 1994, 2014), along with others (Rottinghaus et al., 1993; Saiga, 1998; Miyazaki, 2004), has conducted studies on threshold levels of alkaloids associated with tall fescue toxicosis. Moreover, these studies have been related to clinical signs as seen in fescue toxicosis (Brendemuehl et al., 1995; Blodgett, 2001; Tor-Agbidye et al., 2001; Marin et al., 2013). In contrast, few studies have been done on ergot-derived alkaloids to establish a threshold in livestock for ergotism. Thus, this article discusses the known correlations between the ergot alkaloids and development of ergotism in clinical cases to serve as a starting point for suggesting threshold levels and metabolism of these toxicants.

## Methods

### Extraction of the ergot alkaloids

A method for extraction of ergot alkaloids from plant material was developed based on previous studies, for subsequent analysis by HPLC-fluorescence (Rottinghaus et al., 1991; Hill et al., 1993; Craig et al., 1994; Duringer et al., 2012). A standard mixture comprised of ergonovine, ergosine, ergotamine, ergocornine, α-ergocryptine, and ergocristine was purchased from Romer Labs, Inc. (Union, MO, USA). Seed, straw, hay, or feed pellet samples were ground in a Cyclotec 1093 sample mill and passed through a 0.5-mm screen. To each tube, one gram of sample, 11 mL of chloroform and 1 mL of 0.001 M NaOH were added and mixed for 18–24 h in the dark, then centrifuged at 650 × *g*. Five milliliters of supernatant was applied to a 500 mg/6 mL solid phase extraction (SPE) column containing 0.5 g Ergosil® and 0.5 g anhydrous sodium sulfate. The ergot alkaloids were extracted by adding 5 mL eluent to the SPE, followed by a 2 mL 4:1 acetone:chloroform (v/v) wash and elution with 2.5 mL methanol. The eluent was dried under nitrogen at 50°C, then reconstituted in 0.5 mL methanol. Samples were mixed for 10 s, sonicated for 10 s, and centrifuged at 650 × *g* for 5 min then analyzed via HPLC-fluorescence.

### HPLC-fluorescence analysis for ergot alkaloids

Reverse-phase HPLC analysis is coupled with fluorescence detection (excitation and emission wavelengths of 250 nm and 420 nm, respectively) and a gradient run at 0.9 mL/min. Mobile phases of 1 mM ammonium carbonate (A) and ACN (B) were programmed as follows: equilibrate from 0 to 5 min at 75% A, then decrease linearly to 65% A from 5 to 15 min, hold at 65% A from 15 to 20 min, then decrease linearly to 25% A from 20 to 25 min. A Gemini 3.0 μC18 110Å (Phenomenex, Torrance, CA) column was used in conjunction with a guard column cartridge of similar packing. Figure 1 shows an example of a HPLC-fluorescence chromatogram for ergonovine, ergosine, ergotamine, ergocornine, α-ergocryptine, and ergocristine produced using this assay. [Ergonovine and the epimers of the other ergot alkaloids are not retained by the Ergosil® SPE columns (reference Krska et al., 2008) and are not included in reporting the total ergot alkaloid content of the feed sample]. The method performance has a limit of detection (LOD) of 11 ppb for ergosine, ergotamine, and ergocornine, 13 ppb for α-ergocryptine, and 14 ppb for ergocristine. The limit of quantitation (LOQ) is 39 ppb for ergosine and ergotamine, 41 ppb for ergocornine, 49 ppb α-ergocryptine, and 50 ppb for ergocristine. Within day/Day to day variations are 8.5%/6.5% for ergosine and ergotamine, 4.5%/6.9% for ergocornine, 5.8%/7.8% for α-ergocryptine, and 6.8%/10.6% for ergocristine. The recovery for the above-mentioned alkaloids is 92–97%.

## What we know about vasoconstriction and abortion from ergot alkaloids

Using the data presented in Klotz et al. (2006, 2007, 2010, 2011), we have constructed Table 1, which summarizes the vascular potency and efficacy of ergovaline (the predominant ergot alkaloid found in tall fescue), ergotamine, ergonovine, ergocristine, ergocornine, ergocryptine, and lysergic acid in relative terms. Table 1 bridges what is known in tall fescue toxicosis, i.e., vasoconstriction and the extrapolated abortogenic sequelae. It should be noted that the principal column evaluated in determining the onset of vasoconstriction in clinical disease is column two [Onset (M)].

As Table 1 emphasizes, ergovaline and ergotamine have equal sensitivity for vasoconstriction, whereas ergonovine, ergocristine, and ergocornine are about one-tenth as powerful. Pharmacologically, the half-maximal concentration (EC_50_) and the onset concentration are measures of a compound's potency (the lower the concentration the more potent the compound); ergotamine and ergovaline are identical in this aspect. The onset of vasoconstriction is reported in concentration (moles/L), and is viewed as the best indicator of toxicosis when evaluating levels in feedstuffs. Interpreting the chromatogram in Figure 1 in relation to the additive effects of all ergot alkaloids present is performed by utilizing a multiplicative factor in front of various alkaloids; therefore, 10 ergonovine molecules are equally vasoconstrictive to one ergovaline molecule. More recently, Klotz et al. (2013) has demonstrated that the EC_50_ for ergovaline, ergotamine, and ergocornine can be reduced by an animal's prior exposure to ergot alkaloids. This may have an influence on the animal's perceived sensitivity (as reported by the clinician/researcher) and may affect the threshold of an individual animal (Pesqueira et al., 2014). Further, severe winters and wet springs (wet and cold weather patterns in general) have been shown to be more conducive to ergot-related disease (Welty et al., 1994). This strong correlation between weather and ergot disease is easily seen throughout historical events and in our own clinical cases below (Table 2).

**Table 2:** **Clinical evaluation of ergot toxicosis in cattle with ergotamine equivalence levels (ppb) in feed on a dry weight basis**.

**Case number**	**Total ergot level observed (ppb)^a^**	**Ergotamine equivalence level (ppb)**	**Weather conditions**	**Clinical signs observed**
a	473	473	−20°C, Canada, February	Tail loss
b	1500	415	1°C, Oregon, December	Moderate lameness
c	2909	466	−2°C, Idaho, January	Decreased feed intake
d	3555	778	−5°C, E. Oregon, February	Early term abortions, low milk yield
e	5999	626	−5°C, Idaho, January	No feed consumption
f	11,538	1161	−4°C, Canada, April	Sloughing of hooves
g	54,916	3728	1°C, Oregon, January	Early term abortions
h	62,245	10,124	−1°C, Idaho, January	Hooves sloughing completely off

a *Includes ergonovine, ergosine, ergotamine, ergocornine, α-ergocryptine, and ergocristine*.

## Correlation of ergot toxins with clinical disease

The alkaloids that cause fescue foot and summer slump syndrome are produced by fungi in pasture grasses. Because of similar vasoconstrictive abilities of ergovaline and ergotamine, we have correlated some of the studies done with fescue with what we have seen from clinical cases with ergot. Field and barn studies of natural fescue foot and herds have been conducted (Oliver, 2005; Marin et al., 2013). Therefore, we are illustrating typical cases seen in feed sample submissions over the last 2–3 years to the ESL in Oregon.

In Table 2, column 2 represents the total alkaloids observed, which include a summation of all six ergot alkaloids. Since different alkaloids have different vasoconstrictive onset values, column 3 relates the six alkaloids (Table 1) back to “ergotamine equivalents.” Therefore, ergotamine equivalents are correlated to clinical signs (column 5).

Eight examples of clinical cases associated with feed samples submitted to the ESL by veterinarians suspecting ergot toxicosis are shown to illustrate typical cases that have been seen. The ESL consults with veterinarians and evaluates all aspects of the case to determine if testing of feed samples is warranted. If so, results are often discussed with the client to ensure that proper measures are taken to resolve ergot toxicosis. Five to ten years ago the ESL would observe ergot cases approximately once per month; however, this has progressed to several cases per week and sometimes multiple cases each day in recent years despite maintaining approximately the same number of total samples tested. Our worst toxicosis cases have been in cattle and horses. The increase in cases seen in the Pacific Northwest and throughout the country is likely due to climate change or the lack of field burning that has been phased out throughout the years. The stoppage of burning has greatly reduced the ability to destroy *C. purpurea* by fire.

Evaluation of the cases included in Table 2 is as follows:

The following cases in cattle involved pellets as nearly 100% of the provided feed source, with the exclusion of case b, which used a grass seed sample. Pellets were usually a combination of grass seed with additional alfalfa or other hay sources mixed in. In cases f and h, cattle had exposure to overgrazed pasture, and it is estimated that little nutrition came from the pasture sources. In both cases, the pellet mixture comprised most of the usable diet.

The first case (a) presents an ergotamine level of nearly 500 parts per billion (ppb). This case originated in February when the colder weather prompted vasoconstriction in the extremities, specifically, the tissue around the tail to become necrotic. No sloughing of ears were observed nor reported in this particular case.

The second case (b) reported 1500 ppb ergot alkaloids and occurred in December as the feed bunks were being cleaned out. There was moderate lameness and some necrosis about the feet. The 1500 ppb consisted of ergotamine and ergocornine, resulting in a lower ergotamine equivalence level (415 ppb) that was similar to case (a).

The third case (c) revealed approximately 2900 ppb ergot alkaloids in pellets which triggered a decreased consumption in feed, as well as a necrotic area around the coronet band of the hoof. This outcome was observed in steers in January. Ergotamine, ergocornine, and ergocryptine were found in the feed. The ergotamine equivalence level (466 ppb) illustrated similarities to that of case (a).

The fourth case (d) presented nearly 3500 ppb ergot alkaloids and took place in February. This case showed greater severity of clinical signs, i.e., term abortions, agalactia, and reduced milk production in the dairy herd. This case had a 778 ppb ergotamine equivalence level.

The fifth case (e) presented itself in January and had a total ergot alkaloid concentration of 6000 ppb. The animals refused to consume any feed, resulting in near starvation. This case was composed of very little ergotamine and mostly other ergot alkaloids, which dramatically brought the ergotamine equivalence level down to 626 ppb.

The sixth case (f) occurred during April, with ergot alkaloid levels measuring almost 12,000 ppb. The steers exhibited sloughing of the hooves, tails, and ears. So many animals sloughed their hooves that approximately 40% of the herd had to be terminated. This case consisted of multiple ergot alkaloids and had an ergotamine equivalence level of approximately 1000 ppb.

The seventh case (g) was the second highest case of total ergot alkaloids (54,916 ppb) that we have observed and occurred in January. Though the weather was fairly warm and dry, there were early term abortions in a high percentage of the cow-calf operation. Approximately 45–47 of the 59 animals that were pregnant had early term abortions over a 2 week period. This case had an approximate 4000 ppb ergotamine equivalence level.

The eighth (h) and final case entailed the observance of cattle during January in the northern region of the United States. This case produced approximately 62,000 ppb of total ergot alkaloids. The clinical consequences of the ergot alkaloids resulted in 3/4 of the cow's hooves to slough off, after which the animals had to be sacrificed. This case consisted of both ergotamine and other ergot alkaloids, and had an ergotamine equivalence level to approximately 10,000 ppb. Although the weather was warm, the high concentration of ergot alkaloid induced enough vasoconstriction to clinically affect the animals.

The bioavailability and pharmacokinetic data of ergot alkaloids in cattle are limited and affected by many different factors. However, using the highest and the lowest dietary ergotamine concentrations reported in Table 2, calculations were done to illustrate a theoretical relationship with the ergotamine concentration in a feedstuff and a maximum potential concentration in blood. A feedstuff containing the lowest concentration of 473 ppb (case a) or 0.473 mg/kg ergotamine fed to a 500 kg cow consuming 2.5% of BW will consume a total of 5.9 mg of ergotamine per day. Assuming that the ergotamine is 100% bioavailable from the feedstuff (not always the case as this is negatively correlated with the maturity of the feedstuff), 100% absorbed (not always the case, as these compounds are likely biotransformed by gut microbes prior to absorption), 100% bioavailable in the blood (assuming negligible bioaccumulation in tissues or initial hepatic detoxification) and total blood volume of 55 mL/kg of BW, a maximum ergotamine concentration ever reaching the blood would be 0.215 μg ergotamine/mL of blood. Although this is far below the EC_50_ reported for ergotamine in Table 1 of 4 × 10^−6^ M or an approximate of 2.32 μg of ergotamine/mL in blood, it is much higher than the corresponding onset concentration for ergotamine of 1 × 10^−8^ M or 0.005 μg /mL. If we substituted the highest reported ergotamine concentration in Table 2 (case h), the maximum concentration of ergotamine that could ever reach the blood is 4.6 μg /mL. This is twice the concentration of the EC_50_ of ergotamine and could easily explain the severe clinical signs observed.

The data that resulted from these clinical cases illustrate that ergot alkaloids can cause vasoconstriction and deleterious consequences regardless of seasonal conditions (Egert et al., 2014). Severe vasoconstriction will cause the hooves to slough off and/or abortions. However, in cooler or freezing weather, the values for the vasoconstrictiveness of the different ergot alkaloids are not dissimilar from what is found in endophyte-infected tall fescue with the predominant ergot alkaloid ergovaline (Duringer et al., 2012; Foote et al., 2012). This observation should be documented in future scientific studies. As for now, there have been no specific studies looking at individual alkaloids other than the physiometry values documenting vasoconstriction. Furthermore, there have been no studies related to the convulsive aspect of this disease (neurological and abortogenic) and the individual alkaloids. The only clinical evaluation conducted has been by the papers from Klotz et al. (2006, 2007, 2008, 2010). These papers examine the onset of vasoconstriction. Threshold levels have yet to be established for different animal species. When considering the international transportation of feed, ergot levels become an important factor to evaluate in developing Food Safety standards and programs. In particular, feed produced in the European continent and transported to the Middle East are susceptible to *C. purpurea* due to the wet springs and summers that promote fungal growth.

## What should be done in future studies?

Though this disease has been known for hundreds of years, only a paltry amount of information is available to accurately diagnose disease at a clinical level (where animal production is affected and animal welfare becomes a concern). The first step in expanding this information should be establishing the threshold level in different species, particularly cattle and horses that could be exposed to ergot alkaloids in their feed. It should be noted that overseas, camels, goats, and sheep are also frequently exposed to these toxic alkaloids. The second step is to establish other bio-indicators of clinical disease other than prolactin levels, weight gain, and some of the more classical animal observances. Lastly, the vasoconstrictive and convulsive effects and their subsequent clinical consequences need to be separated.

### Conflict of interest statement

The authors declare that the research was conducted in the absence of any commercial or financial relationships that could be construed as a potential conflict of interest.
